# Advanced multivariate data analysis to determine the root cause of trisulfide bond formation in a novel antibody–peptide fusion

**DOI:** 10.1002/bit.26339

**Published:** 2017-06-05

**Authors:** Stephen Goldrick, William Holmes, Nicholas J. Bond, Gareth Lewis, Marcel Kuiper, Richard Turner, Suzanne S. Farid

**Affiliations:** ^1^ Department of Biochemical Engineering, The Advanced Centre of Biochemical Engineering University College London Gordon Street WC1H 0AH London United Kingdom; ^2^ MedImmune Granta Park Cambridge CB21 6GH United Kingdom

**Keywords:** multivariate data analysis, mammalian cell culture, trisulfide bond, partial least squares modeling, multiple linear regression modeling, product‐related variant

## Abstract

Product quality heterogeneities, such as a trisulfide bond (TSB) formation, can be influenced by multiple interacting process parameters. Identifying their root cause is a major challenge in biopharmaceutical production. To address this issue, this paper describes the novel application of advanced multivariate data analysis (MVDA) techniques to identify the process parameters influencing TSB formation in a novel recombinant antibody–peptide fusion expressed in mammalian cell culture. The screening dataset was generated with a high‐throughput (HT) micro‐bioreactor system (Ambr^*TM*^ 15) using a design of experiments (DoE) approach. The complex dataset was firstly analyzed through the development of a multiple linear regression model focusing solely on the DoE inputs and identified the temperature, pH and initial nutrient feed day as important process parameters influencing this quality attribute. To further scrutinize the dataset, a partial least squares model was subsequently built incorporating both on‐line and off‐line process parameters and enabled accurate predictions of the TSB concentration at harvest. Process parameters identified by the models to promote and suppress TSB formation were implemented on five 7 L bioreactors and the resultant TSB concentrations were comparable to the model predictions. This study demonstrates the ability of MVDA to enable predictions of the key performance drivers influencing TSB formation that are valid also upon scale‐up. Biotechnol. Bioeng. 2017;114: 2222–2234. © 2017 The Authors. Biotechnology and Bioengineering Published by Wiley Periodicals, Inc.

## Introduction

Biopharmaceutical manufacturing utilizing mammalian cell culture expression systems has seen unprecedented growth in the last two decades. This growth has been facilitated by the generation of robust and high‐yielding cell lines, application of scale‐down mimics and high‐throughput technologies, increased process understanding and development of standardized platforms. These advancements have enabled a reduction on the emphasis of maximizing titre and shifted focus onto reducing product development timelines (Bareither and Pollard, 2011; Shukla and Thömmes, 2010) while ensuring consistent product quality profiles (Lubiniecki et al., 2011; Pacis et al., 2011; Zhou and Kantardjieff, 2013). The FDA recommends an adoption of a quality by design (QbD) risk‐based approach for evaluating and mitigating any product‐related variants associated with therapeutic proteins that affect their safety or efficacy (FDA, 2004, 2009, 2014). Typically in an industrial environment the critical process parameters (CPPs) and the critical quality attributes (CQAs) are identified through application of scale down mimics. The challenge of implementing this strategy involves analyzing large volumes of data often collected at different scales and across multiple equipment (Looby et al., 2011). Univariate or time‐series analysis of these complex cell culture datasets can be time‐consuming, inefficient, and lead to misleading conclusions if interactions between variables exist (Kourti, 2006). Therefore, this paper investigates the potential of multivariate data analysis (MVDA) to fully exploit cell culture data and evaluate the key parameter interactions adversely impacting cell culture product heterogeneity.

In biotherapeutic drug manufacturing, there are multiple classes of product‐related variants influencing a drug's functional properties including higher order structure modifications (e.g., misfolding, aggregation), process‐related impurities (e.g., host cell proteins, media components, viral or DNA particles), and post‐translation modifications (e.g., glycosylation, oxidation, glycation, cysteine‐related variants) (Beck et al., 2012; Zhou and Kantardjieff, 2013). Monitoring and controlling product‐related variants are vital to ensure consistent drug safety and efficacy. Recently there have been numerous reports describing monoclonal antibody heterogeneities related to trisulfide bonds (TSBs), defined under cysteine‐related variants (Gu et al., 2010; Kita et al., 2016; Kshirsagar et al., 2012; Liu and May, 2012; Nielsen et al., 2011; Pristatsky et al., 2009). Trisulfides are typically formed through the insertion of a sulfur atom into a disulfide bond within the inter‐chain linkages between the light and heavy chains of the antibody structure. The TSB modifies these linkages, which are essential to ensure the stability of the antibody and can subsequently influence its biological functionality and activity (Kita et al., 2016).

Regulatory approval for process commercialization requires evaluation of parameters on process performance and product quality (Kirdar et al., 2007; Li et al., 2010). Therefore, early stage investigation of product quality issues is essential to ensure accelerated process development timelines. These product heterogeneities can be influenced by environmental changes including time‐series fluctuations of variables such as dissolved oxygen, pH, and temperature (Bareither and Pollard, 2011;Gomez et al., 2010;Kantardjieff and Zhou, 2013). Therefore multifactorial statistical experimentation involving both on‐ and off‐line process adjustments are essential to investigate the potential complex interactions affecting product quality attributes.

To facilitate these multifactorial experiments, industry has invested heavily in the application of high‐throughput (HT) micro‐bioreactor systems that have been extensively demonstrated to replicate laboratory and large‐scale systems (Bareither and Pollard, 2011; Lewis et al., 2010; Long et al., 2014; Micheletti and Lye, 2006; Rameez et al., 2014). However, as these systems are expected to evolve and improve in the coming years (Łącki, 2014), the current challenge involves the ability of the end‐user to effectively consolidate and analyze the high volume of data generated. Thus the application of MVDA has the potential to enhance the analysis of these complex datasets by unveiling hidden process characteristics influencing system performance (Le et al., 2012). In cell culture modeling there are numerous MVDA techniques available. Multiple linear regression (MLR) models and principal component analysis (PCA) studies are highly suited for media optimization (Beg et al., 2003; Liu et al., 2006), scale‐up comparisons (Tsang et al., 2014), and process optimization and development studies involving DoE experimentation (Holmes et al., 2009; Siva et al., 2015). Partial least squares (PLS) modeling is highly suited to process analytic technology (PAT) applications with numerous studies highlighted in the following reviews: Rathore et al. (2010), Glassey et al. (2011), Read et al. (2010) and Simon et al. (2015). More advanced PLS and PCA modeling studies involving on‐line monitoring and statistical control applications were originally demonstrated in the chemical and process‐related industries (Kourti et al., 1995; Nomikos and MacGregor, 1994). These techniques have been successfully applied to bioprocesses in process monitoring (Chiang et al., 2006; Lee et al., 2004; Lennox et al., 2001), fault detection (Gunther et al., 2007), and advanced control applications (Konakovsky et al., 2016).

In this present study, the root cause in terms of process parameters of TSB formation detected in a novel antibody–peptide fusion protein was investigated utilizing advanced multivariate data analysis methodologies. Previous literature has shown that TSB modifications did not influence the pharmacokinetics or biological function of a number of therapeutic proteins (Gu et al., 2010; Kshirsagar et al., 2012; Thomsen et al., 1994). However, in the present work the modification associated with the TSB altered the potency and physicochemical properties of the molecule, even at low levels. Therefore, a thorough investigation that initially looked at the influence of cell line selection and subsequently the bioreactor environmental conditions influencing these high levels of TSB in the molecule of interest was required. The TSB was originally detected during the cell line selection campaign during early pre‐clinical development. The TSB concentration was found to vary between different clones. Therefore, the process parameters influencing this quality attribute required an in‐depth investigation. High levels of TSBs were measured in a subsequent robustness analysis study that explored bioreactor operating conditions beyond the standard range adopted to support early clinical studies. Previous reports have related the presence of hydrogen sulfide in the feeds or cell culture environment to TSB concentration (Kshirsagar et al., 2012; Pristatsky et al., 2009) but little research has been performed on the influence of set‐point changes such as temperature and pH on TSB formation. The focus of this paper is to determine the primary environmental process parameters of the cell culture that either promote or suppress TSB formation enabling process set points to be manipulated to minimize TSB levels at point of harvest. To evaluate these parameters, a three‐level fractional factorial DoE study was undertaken on the Ambr^*TM*^‐15 system manipulating the initial conditions (seeding density), process set‐points (temperature and pH), and feeding strategies (feed day and feed volume).

## Materials and Methods

### Cell Line and Culture Propagation

For the micro‐bioreactor system, a recombinant Chinese hamster ovary (CHO) cell line expressing high levels of an antibody–peptide fusion was used. The cell line was cultured in animal component‐free chemically defined CHO media. The cells were maintained at 37°C under 5% carbon dioxide, shaken at a constant rpm, and passaged 2–3 times per week for propagation and scale‐up for inoculation. The same cell line and inoculum protocol was used for the 7 L bioreactor experiments.

### Bioreactor Systems

The screening experiments were conducted using a DoE approach with a micro‐bioreactor (Ambr^*TM*^‐15) system (TAP Biosystems, Greenville, DE) with 48 single vessels split into four separate culture stations where each vessel was operated with a 11–15 mL working volume. The temperature and pH of each culture station was controlled as described by the DoE in Design of Experiments Conditions section and the agitation rate was kept consistent for each culture station. Similarly, the feeding strategy was manipulated for each individual vessel as described by the DoE in Design of Experiments Conditions section. Off‐line daily samples were taken from each vessel. Further detailed descriptions of the Ambr^*TM*^‐15 system can be found in Rameez et al. (2014). The experiments performed in the 7 L bioreactor (Applikon Biotechnology, Tewkesbury, Gloucestershire, UK) were carried out with a working volume of approximately 5 L.

### Cell Culture Process

In the Ambr^*TM*^‐15 system, the cell culture temperature and pH were maintained at set‐points indicated by the DoE in Design of Experiments Conditions section where the mid‐point of each variable represents normal operating condition. The initial seeding density indicated by 1 in the DoE represents the seeding density of <10 × 10^5^ cells/mL for normal operation. Similarly, the nutrient feed volume equal to 1 represents the normal operating volume of the proprietary nutrient feed. The feeding strategy involved five equally spaced additions of the feed after the initial feed day indicated by the DoE design. The dissolved oxygen set point was set to 50% in all culture stations and was maintained by gassing with air and oxygen and manipulation of the agitator RPM. The agitator speed for all culture stations was systematically ramped up throughout the cell culture operation to ensure the dissolved oxygen set‐point was maintained, the RPM adjustments were consistent for all four culture stations. The culture pH was controlled through the addition of sodium carbonate and sparging CO_2_ gas with its control strategy implementing a pH dead‐band equal to 0.1. Antifoam was added as required. Daily at‐line samples were analyzed for viable cell concentration (VCC) and viability using the Vi‐Cell Automated cell viability analyzer (Beckman Coulter, Brea, CA), and glucose and lactate were analyzed using the Bioprofile flex (Nova Biomedical Corporation, Waltham, MA).

In the 7 L cell culture runs, the reactor temperature was controlled using an external electric heating blanket at the temperature set‐point indicated in Table I. The pH set‐points are indicated also in Table I and were maintained using a CO_2_ gas supply and additions of a sodium carbonate solution. The DO_2_ was maintained at a set‐point of 50% controlled by the addition of O_2_ gas and the agitation of each vessel was ramped up as required to maintain the DO_2_ at its set‐point. Off‐line analysis of viable cell density and off‐line samples was conducted as described for the Ambr^*TM*^‐15 system.

**Table I:** Summary of manipulated process parameters for the five 7 L cell culture runs implemented to validate the TSB predictions of the MVDA models at scale

Run ref	*T* (°C)	NFD (day)	NFV (mL/mL)	pH (pH)	SD (cells mL^−1^/cells mL^−1^)
Run‐1	35.5	Day‐2	1	7.00	1
Run‐2	37	Day‐3	1	6.90	0.8
Run‐3	37	Day‐3	1	6.90	0.8
Run‐4	35.5	Day‐3	1.1	6.90	1.2
Run‐5	35.5	Day‐4	1	7.00	1

### Titre Analysis

Volumetric antibody–peptide fusion titres in cell culture supernatants were quantified by protein A affinity chromatography using a protein A ImmunoDetection sensor cartridge (Applied Biosystems, Warrington, UK) coupled to an Agilent 1200 series HPLC (Agilent, Berkshire, UK). Peak areas relative to a reference standard calibration curve were used to calculate titres. These samples were measured on days 8, 10, 12, and 14 for the Ambr^*TM*^‐15 system and on days 4–14 for the 7 L cell culture operation.

### TSB Quantitation: Targeted Mass Spectrometry

The TSB concentrations were quantified at the end of each cell culture run by measuring the proportion of modified and unmodified peptide as determined by selected ion monitoring (SIM) following digestion of the purified protein. TSB concentrations were reported as the proportion of molecules containing a TSB. The modification specific to the TSB was identified by peptide mapping using liquid chromatography mass spectrometry (LC‐MS). Measurements were made on protein A purified samples digested using an automated liquid handler (BRAVO, Agilent Technologies, Santa Clara, CA) followed LC‐MS using an Acquity iClass UPLC and Xevo TQS triple quadrupole mass spectrometer (Waters, Milford, MA). Briefly, protein samples at 5 mg/mL were denatured by incubation in 5 M Urea, 100 mM Tris, pH 7.6 for 30 min at 37°C prior to tryptic digestion (1:10 enzyme/substrate ratio) in 2 M Urea, 100 mM Tris, and pH 7.6 for 4 h at 37°C. Liberated peptides were chromatographically separated over a 6 min gradient using a 150 × 2.1 mm BEH C18 UPLC column (Waters) and flow rate of 0.2 mL/min; mobile phases A and B were water and acetonitrile, respectively, supplemented with 0.02% trifluoroacetic acid. Peptide ions were formed using a source temperature of 600°C, cone voltage of 45 V, and cone gas flowrate of 400 L/h. SIM transitions were used for quantitation and selected reaction monitoring (SRM) transitions used for confirmation of analyte identity.

### Software

All data manipulation and analysis including PLS and MLR model generation were performed using Matlab 2014a (The MathWorks, Inc., Natick, MA).

### Design of Experiments Conditions

The five variables included in the DoE were the temperature set‐point (*T*), initial nutrient feed day (NFD), nutrient feed volume (NFV), pH set‐point (pH), and initial seeding density (SD). A fractional factorial design was chosen using an optimal design of resolution equal to four consisting of 28 runs plus three center points to provide a measure of process stability and inherent variability. Furthermore, to broaden the scope of the design, two axial points for each variable (except temperature) were added, resulting in an additional eight axial points. An additional two axial points at pH 6.85 and 7.05 were investigated at the high and low temperature resulting in an extra four runs in the design. In total this resulted in 43 experiments, and the remaining five culture stations in the Ambr^*TM*^‐15 system were not included in this DoE. The finalized experimental design is summarized as follows:

- Temperature (°C): 3 levels—factorial points: 34, 35.5, 37.
- Initial nutrient feed day (Day): 3 levels—factorial points: 1, 2, 3 with two axial points: 1 and 4.
- Nutrient feed volume (mL/mL): 3 levels 0.9,1, 1.1 with two axial points: 0.8 and 1.2.
- pH (pH): 3 levels 6.9, 7, 7.1 with two axial points: 6.8, and 7.2.
- Initial seeding density (cells mL^−1^/cells mL^−1^): 3 levels 0.8, 1, 1.2 with two axial points: 0.57 and 1.57.

## Computational Methods

### Multiple Linear Regression Model Development

**Figure 1:**
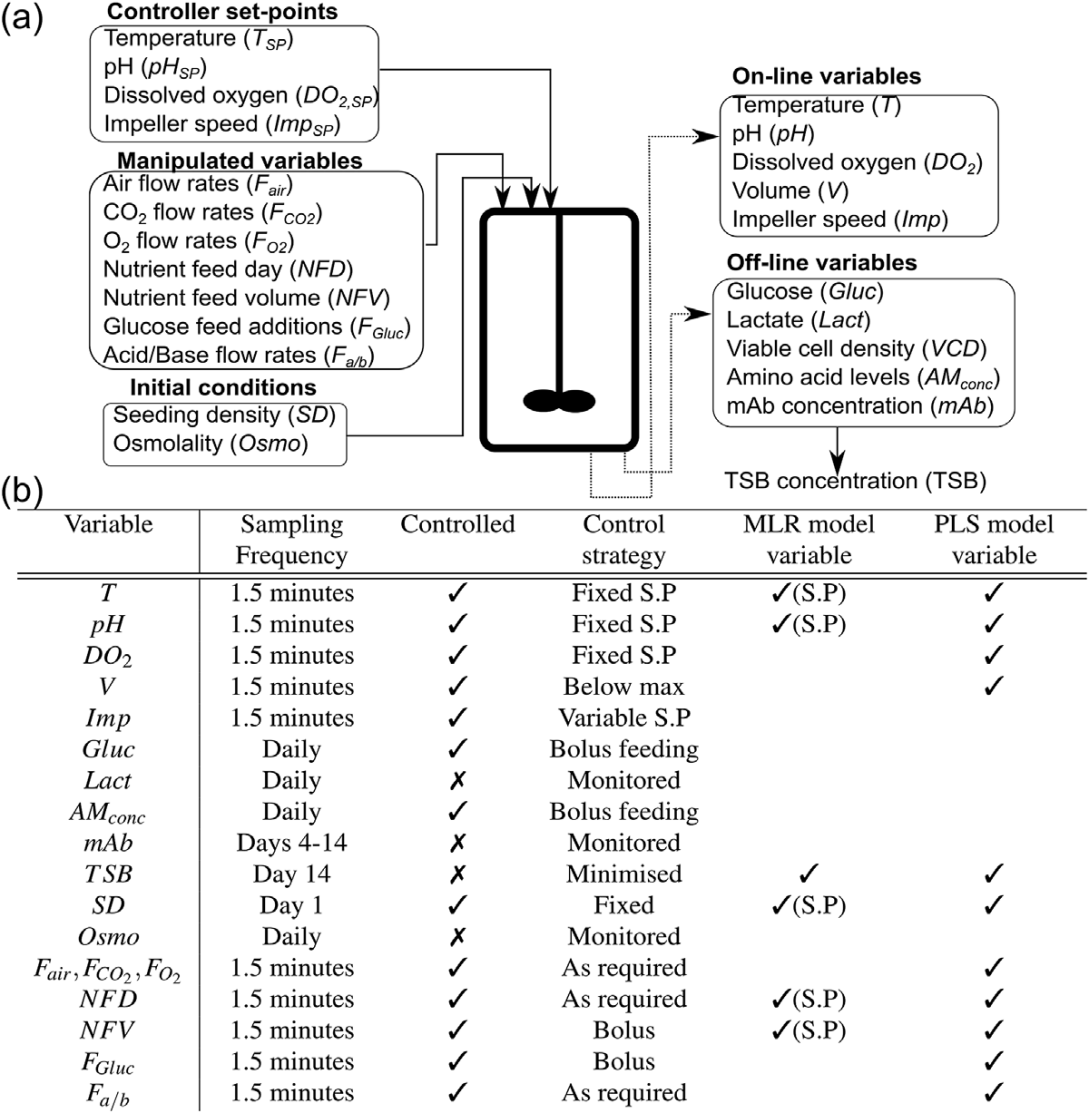
(**a**) Schematic of Ambr^*TM*^‐15 system outlining the different types of variables recorded. (**b**) Embedded table outlining the sampling frequency and control strategy implemented on the micro‐bioreactor system with the variables selected for analysis by the MLR and PLS also highlighted. S.P represents the variable set‐point.

Multiple linear regression (MLR) is a mathematical technique enabling predictions of a single‐dependent variable from multiple‐independent variables. In fed‐batch cell culture systems, the inputs for MLR analysis are typically single time point fixed variables that are representative for the entire culture length and include initial conditions, media formulations, fixed controller set‐points, and feeding strategies. In this present study, the MLR model inputs were the temperature set‐point (*T*), the pH set‐point (pH), the initial nutrient feed day (NFD), the nutrient feed volume (NFV), and the seeding density (SD) with the TSB concentration as the response as indicated in Figure 1. The generated model can quantify the relative importance of the input variables through analysis of the model's regression coefficients and is highly suited to analyze DoE type data. To enable easier interpretation of the model coefficients and allow comparison of each input on a common scale, the MLR model inputs were converted to coded factors.

A quadratic MLR model that assumed interaction between the variables was initially chosen and a stepwise regression approach implementing both forward addition and backward elimination was used to generate the final model. The selection criteria for the finalized model was based on minimizing the root mean square error (RMSE) between the model predictions and the experimental TSB concentrations. The final MLR model generated by this procedure was as follows:

(1) $$[TSB]=\beta_{0}+\beta_{1}T+\beta_{2}NFD+\beta_{3}pH+\beta_{4}TpH+\beta_{5}NFDpH$$

where [TSB] is the predicted TSB concentration with the temperature taken as *T*, nutrient feed day as NFD and the pH as pH. The intercept of the model is taken as *β*_0_ and the coefficients taken as *β*_1,2,…5_.

### PLS Model Development

**Figure 2:**
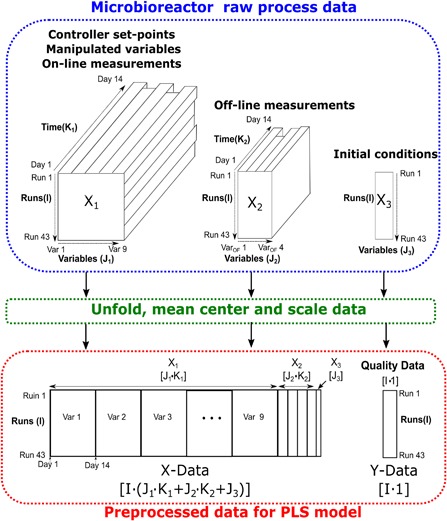
Outline of data consolidation required for the generation of the PLS model involving the unfolding and scaling of the on‐line measurements (*X*_1_ block), off‐line measurements (*X*_2_ block) and initial conditions.

In addition to analyzing discrete datasets, more advanced MVDA techniques are required to analyze both continuous and discrete datasets. PLS is highly suited for the analysis of high‐throughput cell culture data based on its ability to reduce large data matrices into low‐dimensional vector spaces allowing for easier data interpretation and better visualization of hidden correlations. One of the main challenges in applying PLS as a statistical tool for cell culture analysis involves the complicated pretreatment of the various different data structures recorded using multiple devices at varying time frequencies (Nomikos and MacGregor, 1994). Figure 2 highlights the various data blocks recorded by the micro‐bioreactor system employed here, where the *X*_1_ block consists of *i*_1_ = 1,2,3& *I* cell culture runs with *j*_1_ = 1,2,3& *J*_1_ on‐line process or manipulated variables that were recorded at *k*_1_ = 1,2,3& *K*_1_ time intervals forming a three dimensional *I *× *J*_1_ × *K*_1_ block. Similarly, the infrequently recorded off‐line variables are summarized by a *I *× *J*_2_ × *K*_2_ block represented here as the *X*_2_ block with *J*_2_ representing the number of off‐line variables and *K*_2_ represents the sampling time for each variable. The initial conditions are represented by the *X*_3_ block, equal in size to *I* × *J*_3_, where *J*_3_ is the number of initial conditions. The response variable is represented by the *Y* block and in this case is equal to the final TSB concentration of each cell culture run and equal to an *I* × 1 vector. Unfolding these three‐dimensional blocks and consolidating the additional process measurements together is necessary to enable PLS modeling as this technique is only suitable for two dimensional analysis. Wold et al. (1987) extended this technique to multi‐way partial least squares (MPLS), which is equivalent to performing ordinary PLS on a large two dimensional matrix formed by unfolding the three dimensional blocks. In the analysis of cell culture processes, the most meaningful way to unfold the data is by batch‐wise unfolding, where a batch represents a single cell culture run. There are various other ways to unfold the three dimensional batch data that are better suited to model variation between variables and across time intervals (Chiang et al., 2006; Lee et al., 2004). Yet the focus here is on batch‐wise unfolding as it enables investigation of the run‐to‐run variability highlighted in the given problem statement. The cell culture data summarized in the three different blocks was firstly unfolded and consolidated into a new *X* block of size equal to *I *× *L* where *L* is equal to *I* × (*J*_1_ *K*_1_ + *J*_2_ *K*_2_ + *J*_3_) as defined in Figure 2. As standard with PLS data pre‐treatment all variables were mean‐centered and scaled to unit variance by dividing by their standard deviation. Additionally, to ensure the initial conditions containing a single time point were not dominated by the vast number of on‐ or off‐line variables, variable block scaling was incorporated. This ensures each variable was considered equal in importance and weighting in the PLS model and is defined as follows:

(2) $$X=\left[\frac{X_{1}}{\sqrt{K_{1}}}\frac{X_{2}}{\sqrt{K_{2}}}X_{3}\right]$$

To calculate the PLS model parameters, the non‐linear iterative partial least squares (NIPALS) algorithm was implemented (Wold et al., 1987). The PLS algorithm consists of an outer‐relationship that considers the *X* and *Y* blocks individually and an inner‐relationship that links the two blocks together. The outer relationships are generated by decomposing the newly unfolded *X* (*I *× *L*) and *Y* (*I *× 1) blocks into *R* latent score variables [*t*, *u*], loading vectors [*p*, *q*], weights *W* (*L *× *R*), and the model residual matrices *E* (*I *× *L*), *F* (*I *× 1). *t*, *u*, *p*, and *q* can be combined into *T* (*I *× *R*), *U* (*I *× *R*), *P* (*L *× *R*), *Q* (1 × *R*), and *W* (*L *× *R*) as defined below:

(3) $$X=\sum_{r=1}^{R}t_{r}p_{r}^{T}+E\;X={TP}^{T}+E$$

(4) $$Y=\sum_{r=1}^{R}u_{r}q_{r}^{T}+F\;Y={UQ}^{T}+F$$

A vector of inner‐relationships *B* (*R *× *R*) is generated that relates the scores of the *X* block to the *Y* block as follows:

(5) $$B=U^{T}T{\left(T^{T}T\right)}^{-1}$$

The PLS model implements an iterative procedure for each latent variable to reach convergence and once the procedure is complete, a matrix of regression coefficients *β* (*L *× *R*) can be generated as follows:

(6) $$\beta =W{\left(P^{T}W\right)}^{-1}diag(B)$$

where *W* equals (*U* ^T^ *X*)^T^. The cumulative sum of the regression coefficients predicts the response variable ($\hat{Y}$) from the *X* block taking *R* latent variables:

(7) $$\hat{Y}=X\sum_{r=1}^{R}\beta$$

The weights matrix *W* within the PLS model carries valuable information relating the *X* and *Y* blocks. Eriksson et al. (2001) summarized this information in the variable importance on projection (VIP_j_) plot for each variable j considered in the PLS model, calculated as follows:

(8) $$VIP_{j}=\sum_{r=1}^{R}w_{rjk}^{2}(SSR_{r}\setminus SSY_{R})$$

where $w_{rjk}^{2}$ is the squared sum of the weights of each variable j recorded across *k* time‐points for each latent variable r. SSY_r_ is the sum of explained variance of the *Y* block for the *r* ^th^ latent variable and SSY_R_ is the total variance explained by the PLS model taking *R* latent variables.

The variables included in the PLS model are outlined in Figure 1. Each of the variables was unfolded and scaled as previously discussed to form the *X*‐block or *Y*‐block. The *X*‐block data were divided into calibration and validation data sets composed of 33 and 10 batches, respectively. Five latent variables were selected based on minimizing the root mean squared error (RMSE) of the 33 calibration batches and the root mean squared of prediction (RMSEP) of the 10 validation batches. The five latent variables captured 56% of the total variance of the *X*‐block and 95% of the *Y*‐block. Taking additional latent variables unnecessarily increases the complexity of the PLS model with only a marginal decrease in the RMSE and RMSEP.

## Results and Discussion

**Figure 3:**
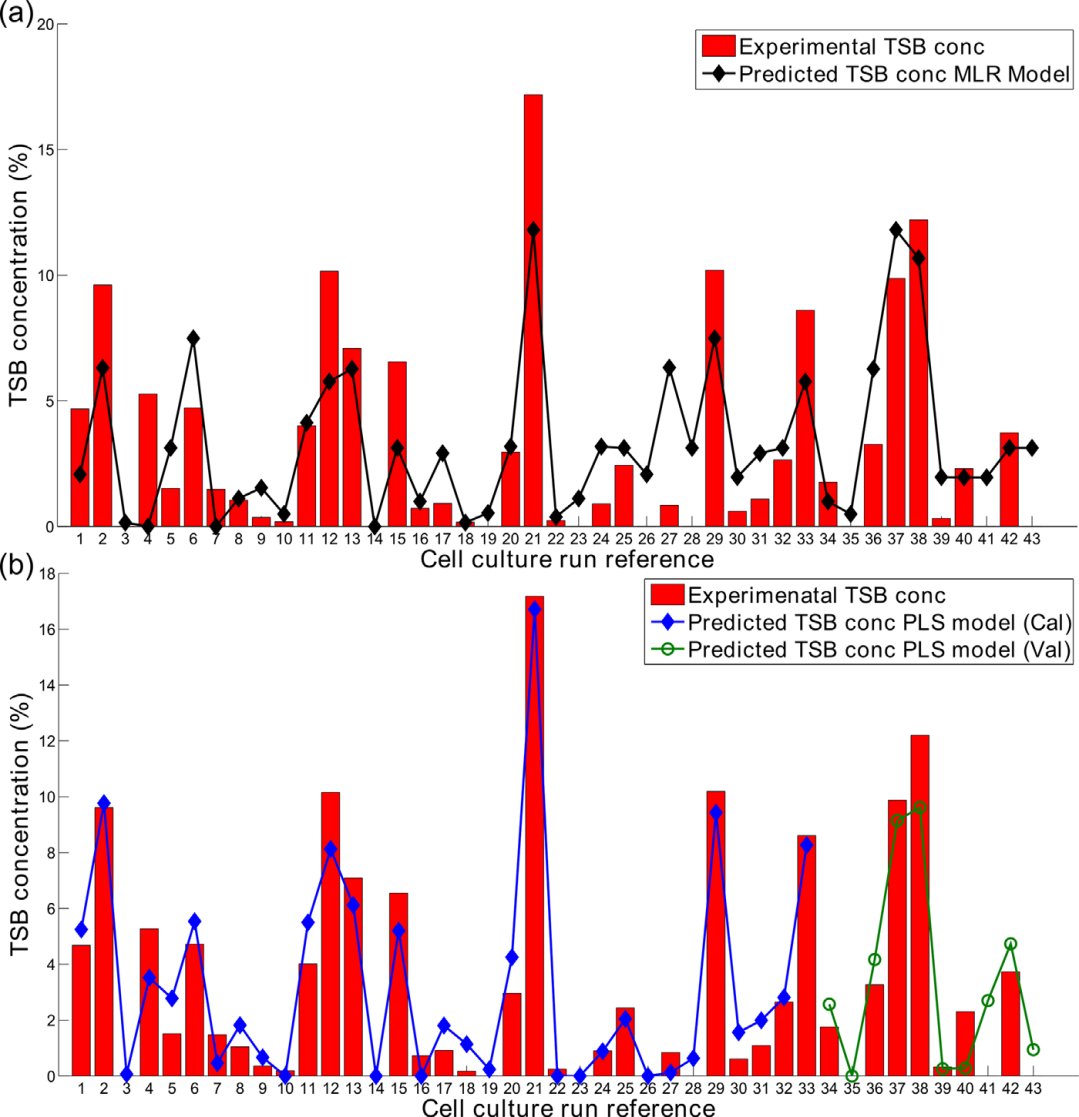
Comparison of predictions of TSB concentrations against experimental values using (**a**) the MLR model (*R* ^2^ equal to 0.68) and (**b**) the PLS model (*R* ^2^ of calibration dataset equal to 0.89 and the *R* ^2^ of validation dataset equal to 0.7).

A screening study using a DoE approach was carried out to determine the root cause of high TSB concentrations detected in an antibody–peptide fusion protein. The variable operating range selected in this study resulted in TSB concentrations varying from 0% up to 17.2% as is shown in Figure 3. These previously unseen high TSB concentrations demonstrated the significant impact of the chosen variable operating range on product quality. Similarly, Gu et al. (2010) reported TSB concentrations for seven different monoclonal antibodies as high as 39.2% and on average equal to 11.5% with a standard deviation of 7.5% demonstrating the significantly high occurrence of TSBs in recombinant proteins.

A multiple linear regression was implemented to assess the influence of the manipulated DoE inputs on the TSB levels. These data were further scrutinized by a PLS model that quantified the impact of both the on‐line and off‐line variables on the detected TSB levels.

### MLR Model Predictions

**Figure 4:**
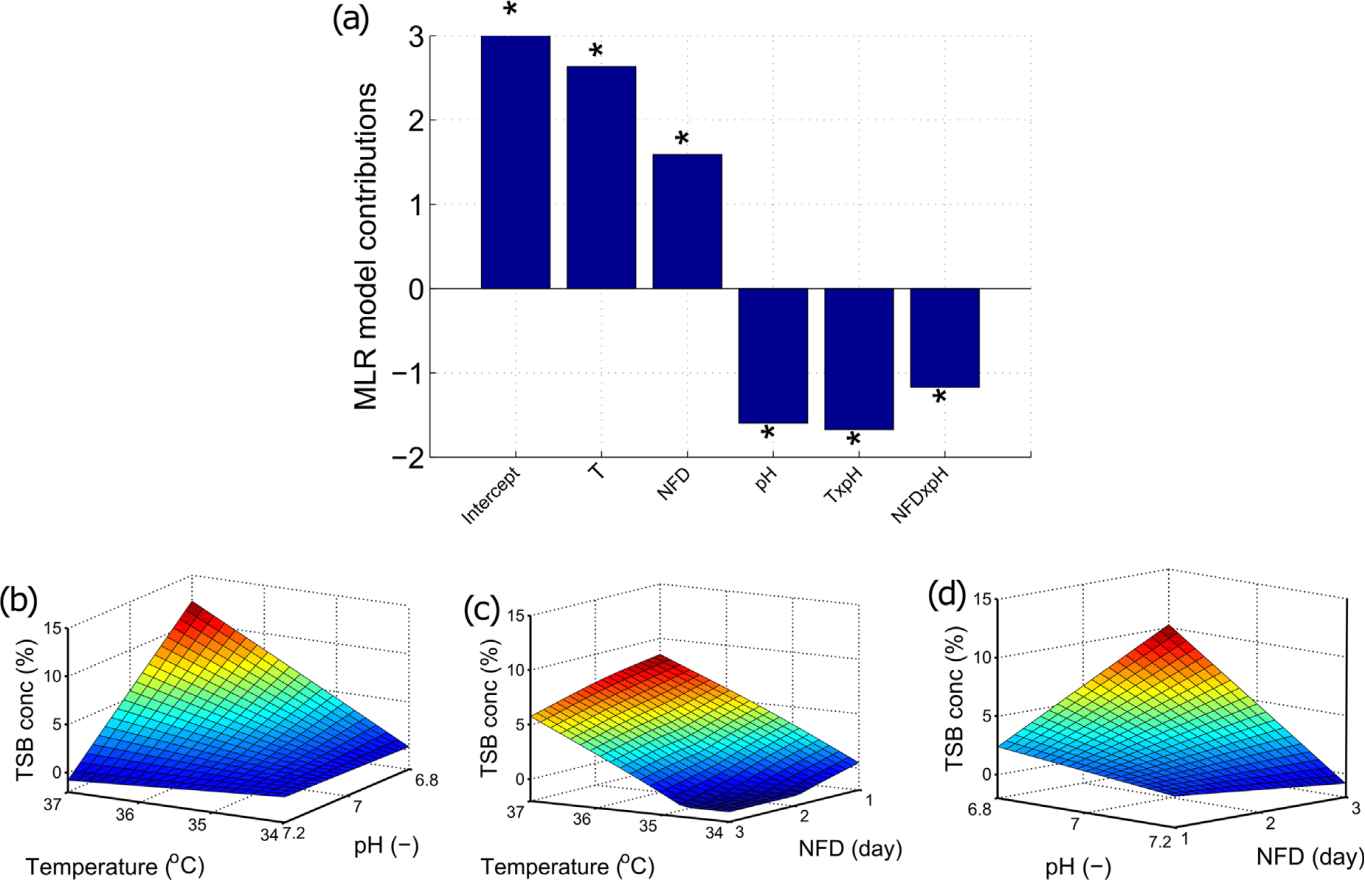
(**a**) Contributions of the MLR model terms used for the prediction of the TSB concentration generated using coded factors. The * indicates the model terms are significant based on a *P*‐value of less than 0.05. Response surfaces highlighting the effects of TSB levels of (**b**) temperature and pH with NFD equal to 2, (**c**) NFD and temperature with pH equal to 7, and (**d**) pH and NFD with temperature equal to 35.5°C.

To quantify the impact of the environmental cell culture changes on the TSB concentrations, the complex dataset was initially analyzed through the development of an MLR model focusing solely on the DoE set‐point changes as discussed in Multiple Linear Regression Model Development section. The MLR model was shown to capture 68% of the total variance of the TSB concentrations and the model predictions were comparable with the experimental recorded values as shown in Figure 3a. Through analysis of the MLR model coefficients, the temperature set‐point, initial nutrient feed day, and pH set‐point were found to be highly influential on the final TSB concentration. Additionally, interactions between temperature and pH and nutrient feed day and pH were also found to be significant. Interestingly, both the nutrient feed volume and initial seeding density did not contribute significantly to the TSB ­levels and were eliminated from the model. All of the terms used in the model were statistically significant based on a *P*‐value of less than 0.05. Sullivan and Feinn (2012) discussed that although the statistical significance of a variable is important, the effective magnitude of each variable on the predicted response is the most important factor to consider. Figure 4a shows the effective magnitude of the MLR model coefficients generated using coded factors allowing for their effective contributions to be interpreted directly. Temperature has the largest influence on the model and the analysis indicates a positive correlation with TSBs, i.e., high temperatures (37°C) promote high TSB concentrations. The main effects of pH and NFD were similar in magnitude but opposite in sign, highlighting a negative correlation. Two interactions were also found to influence the TSB concentration. Figure 4b–d shows the model predictions considering both the main and interactive terms of the MLR model for the variable operating range investigated in this work. The highest predicted TSB concentration, was observed with high temperature (37°C), low pH (6.8), and nutrient feed day set to day 2. Previous reports have also highlighted the significant influence of cell culture operating temperature on TSB formation during antibody production (Evans et al., 2011).

Considering both the main effects and interaction terms, the MLR model suggests that TSB formation is promoted by a high temperature and low pH set‐point operated with a late feeding strategy. Both the seeding density and nutrient feed volume were found to have little influence on this quality attribute. Previously, Kshirsagar et al. (2012) correlated the concentration of cysteine in the feeds to TSB concentrations suggesting an increase in feed on a per cell basis leads to higher TSB levels. In the present study, analyzing the TSB concentration as a function of the feed on a per cell basis resulted in no correlation (data not shown).

### Time Series Fluctuations

**Figure 5:**
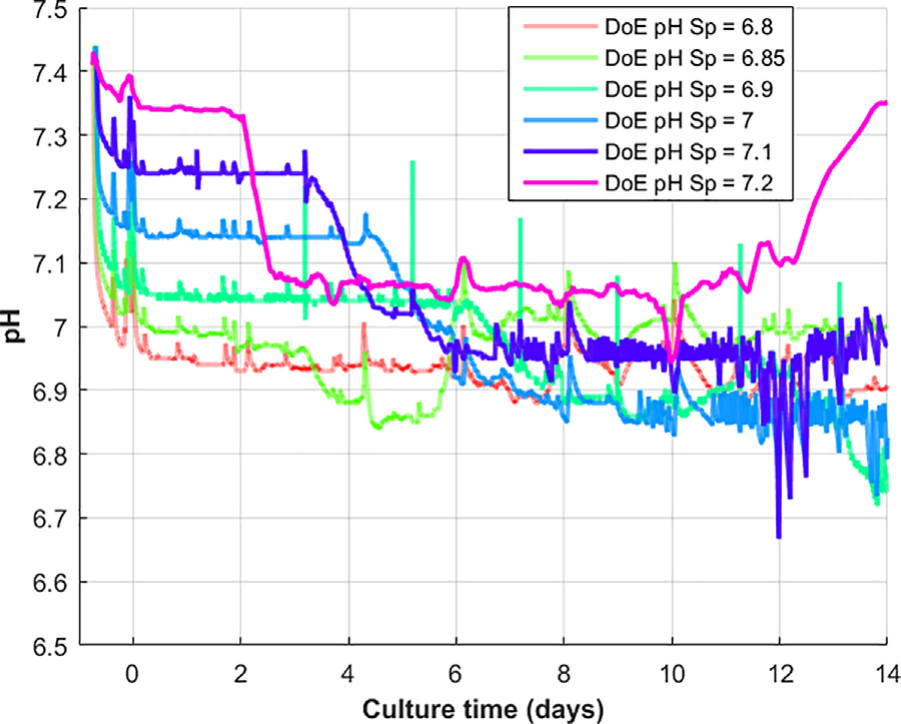
pH profiles of the six different DoE set‐point changes recorded on the micro‐bioreactor system.

One limitation in developing MLR models on cell culture systems is the assumption that discrete model input variables selected by the model can accurately represent the observed process conditions across the entire cell culture length. However, as discussed by Formenti et al. (2014), controlling cell culture systems to their respective set‐points or trajectories is difficult due to the complex non‐linear nature of cell culture systems. For this particular process, the pH dead‐band was set to 0.1 allowing the pH to drift around its set‐point without triggering the addition of base or CO_2_ gas. Therefore, the pH was found to deviate around its set‐point as is highlighted in Figure 5 where the pH profiles of six micro‐bioreactors with different pH set‐points are shown. Previous literature has emphasized the importance of controlling the pH of mammalian cell culture systems to ensure consistent product quality (Borys et al., 1993; Mohan et al., 1993). These profiles suggest the pH set‐points included in the MLR model are not representative of the actual pH recorded experimentally by each micro‐bioreactor. Similarly gas flow rates, impeller speeds, and off‐line variables are continually changing throughout the duration of the cell culture run. Therefore, these dynamic adjustments are not accurately represented by discrete variables. To account for these time‐series fluctuations and further scrutinize the available dataset, PLS modeling was implemented.

### PLS Model Predictions

**Figure 6:**
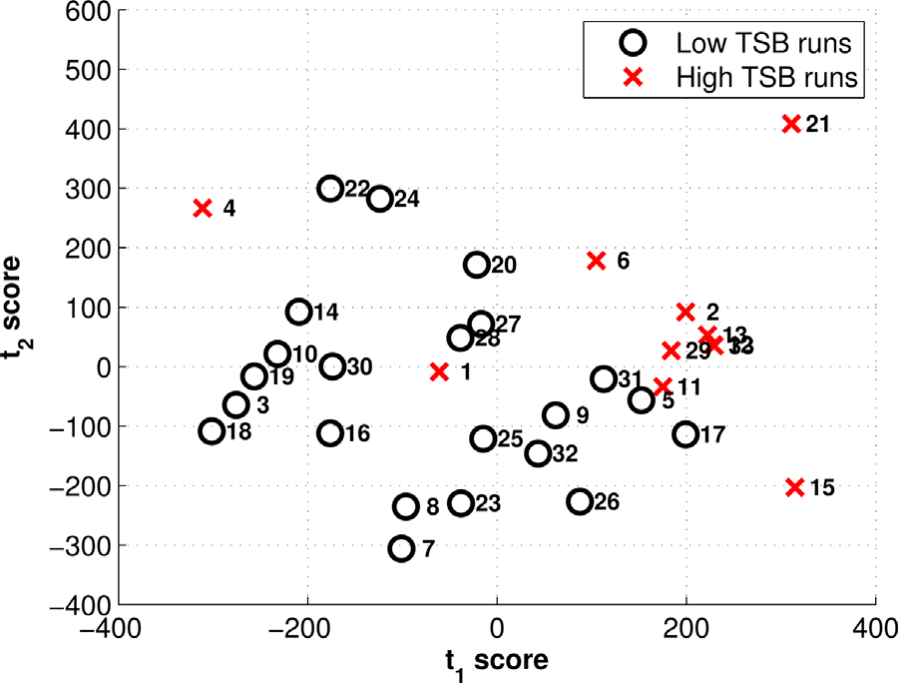
Scores plot of the first two latent variables for the 33 calibration runs where “Low TSB” runs had a final TSB concentration of 3.5% or lower and cell culture runs with higher concentrations were classed as “High TSB” runs. *t*_1_and *t*_2_ represent the scores from the first and second latent variables, respectively.

In addition to capturing time‐series fluctuations, the PLS technique enables a wider range of process variables recorded by the micro‐bioreactor system to be included in the analysis as is summarized in Figure 1. This is particularly relevant considering the continued development of process analytics and the need to exploit the important information from these complex data sets. The PLS model parameters defined in Equations (3) and (4) were used to evaluate the process performance of each run and to identify any discriminating run characteristics to enable root‐cause determination of the TSB concentration levels. The scores of the first two latent variables, *t*_1_ and *t*_2_, accounting for 43% and 18% of the total variance observed in the final TSB concentration, respectively, are shown in Figure 6. A natural clustering of “Low TSB” and “High TSB” cell culture runs is highlighted in Figure 6 where “Low TSB” runs had a TSB concentration of 3.5% or less and runs above this limit were classed as “High TSB” runs. The clustering of the majority of “High TSB” runs indicated similar cell culture characteristics were identified by the variables in the PLS model that are highly correlated with the TSB concentration. To investigate which variables had the largest influence on the TSB concentration, the variable contributions calculated from the regression weights (*β*) of each latent variable were analyzed.

**Figure 7:**
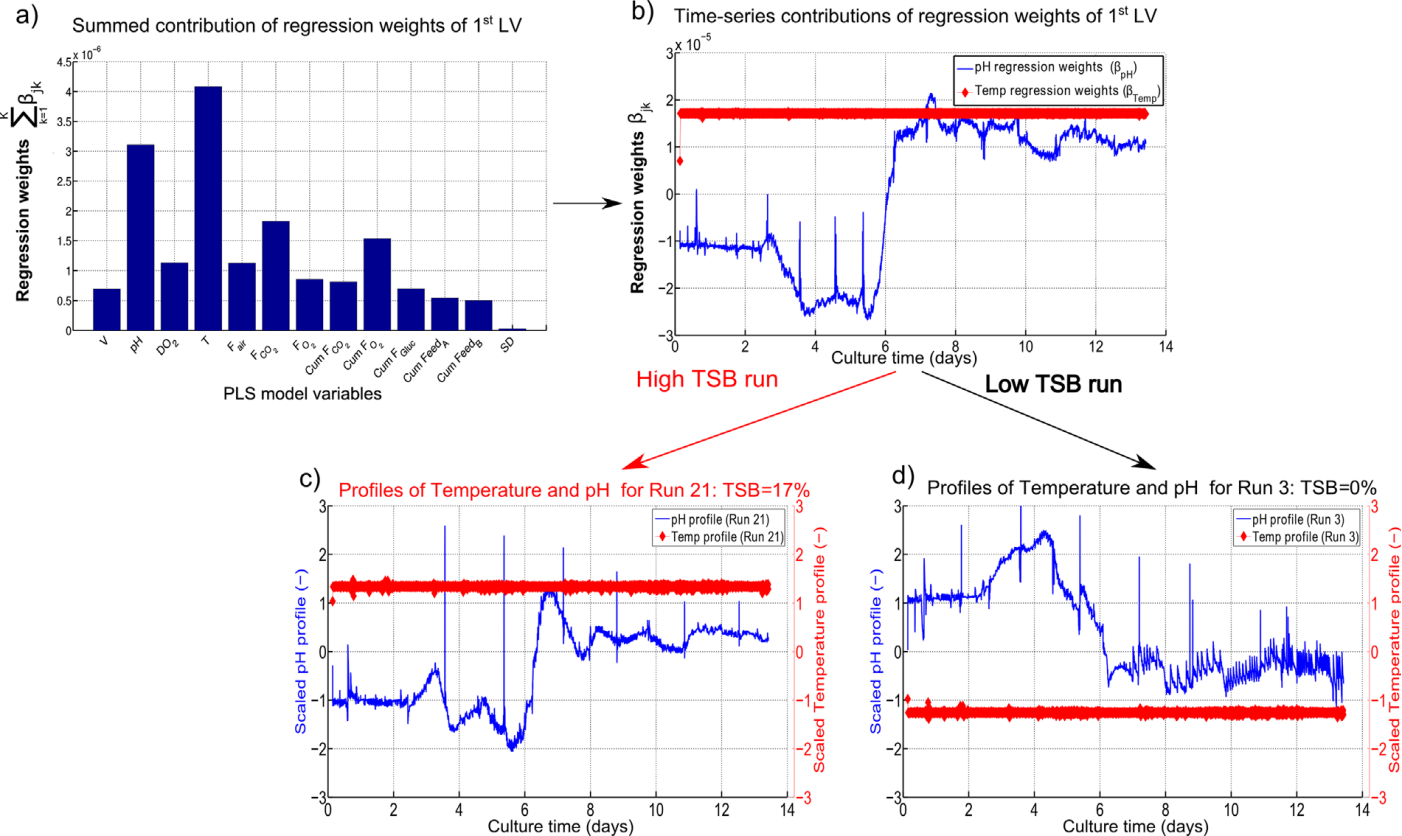
(**a**) Summed regression weights of each variable $\left(\sum_{k=1}^{K}\beta_{jk}^{2}\right)$ with j representing the variables included in the PLS model recorded across its k time points. (**b**) The time‐series regression weights (*β*_jk_) of temperature and pH for the first latent variable of the PLS. (**c** and **d**) show the corresponding temperature and pH profiles of Run‐21 and Run‐3 that had a TSB concentration of 17% and 0%, respectively. *β*_jk_ indicates the time‐series regression weights for the variable (j) recorded across its k time‐points.

The summed contributions $\left(\sum_{k=1}^{K}\beta_{jk}^{2}\right)$ for each variable j recorded across k time points calculated for the first latent variable are shown in Figure 7a, highlighting the significant contribution of both the temperature and pH. However, high variable contributions do not necessarily indicate a direct influence on the prediction of the response variable *Y*. As indicated in Equation (7), the prediction of the final TSB concentration is the product of the regression weights by the scaled time‐series profile of each variable, hence their profiles must be considered simultaneously to ascertain their influence on the predicted response variable. The regression weights for the temperature (*β*_T_) and pH (*β*_pH_) are shown in Figure 7b. The relatively constant profile observed for *β*_T_ indicates the variable does not fluctuate whereas the significant fluctuations observed for *β*_pH_ indicates the pH deviates throughout the cell culture run.

To correctly interpret these weights, the scaled pH and temperature profiles must be taken into account. The profiles of these variables are shown for two runs, Run‐21 and Run‐3 that had a final TSB concentration of 17.2% and 0%, respectively, and can be considered as an example of a “High TSB” and “Low TSB” run. Examination of the temperature weights (*β*_T_) shown in Figure 7b and the temperature profile of the “High TSB” run in Figure 7c highlights that the product of these two matrices would result in a high TSB value as both are positive in magnitude across the entire length of the cell culture run. The opposite is observed for Run 3 indicating the lower temperature suppresses the formation of TSBs. Thus, the PLS model indicates that high temperatures promote TSB formation whereas low temperatures suppress it. The regression weights generated for the pH (*β*_pH_) are more difficult to interpret based on the large fluctuation observed in Figure 7b. Initially the pH has a negative contribution and after day 6 a sharp transition to a positive contribution is observed. In order to suppress the TSB formation a positive scaled pH up to day 6 followed by a negative pH would negatively contribute to the TSB concentration. Interestingly, this ideal pH profile is very similar to the experimental recorded pH for Run‐3 with a 0% TSB concentration.

In contrast, the pH profile shown for Run 21 in Figure 7c, with a TSB concentration of 17%, is similar in shape to the regression weights and therefore its product would result in a high TSB concentration. The experimental pH values of the scaled pH profiles shown in Figure 7c and d fluctuate between pH 6.9 and 7.1. Interestingly, the pKa values of H_2_S to H^+^ + HS^−^ have been reported to be equal to approximately 7 (Li and Lancaster, 2013). Therefore, the high observed pH (above 7) of Run 21 toward the end of the run would potentially increase the acidic dissociation of H_2_S that could promote a nucleophilic attack of the disulfide bond by the HS^−^ resulting in increased TSB formation as hypothesized by Nielsen et al. (2011). The PLS model suggests a low pH toward the end of the cell culture run could reduce the formation of TSBs based on these pH regression weights. Additionally, the sharp contrast observed between the two pH profiles of Run 21 and Run 3 highlights the potential of this variable to be used an indicator in a batch‐process monitoring strategy to enable fault detections for early predictions of runs with high TSB concentrations.

**Figure 8:**
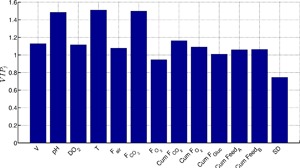
Variable influence of projection (VIP_j_) for PLS model consisting of five latent variable.

Further to analyzing the individual latent variables, it is important to consider the cumulative contribution of the original variables that contribute toward selected latent variables in the PLS model. This can be summarized by the variable influence on projection (VIP_j_) defined by Equation (8). The VIP_j_ plot for the five latent variable PLS model is shown in Figure 8. The threshold for important variables is defined by Tran et al. (2014) and Cinar et al. (2003) to be equal to 1, where “VIP_j_ > 1” indicates the variable j is highly influential on the TSB concentration. The importance of the pH and temperature were discussed previously and their significant contributions are also observed from the VIP_j_ plot. The VIP_j_ plot also highlights the CO_2_ flow rate (*F*_CO2_) as a highly significant variable based on its contribution to the TSB concentration. Analyzing the regression weights of *F*_CO2_ suggests increased CO_2_ gas‐flow rates reduce the formation of TSBs. This observation is similar to the mitigation strategy outlined by Becker and Christensen (2006); they demonstrated that sparging gas during the harvest step of a human growth hormone fermentation resulted in reduced trisulfide concentrations by stripping out the hydrogen sulfide from the vessel.

### Trisulfide Bond Mechanism

Previous reports have demonstrated trisulfides are a post‐translation modification formed by the insertion of a sulfur atom into a disulfide bond due to the presence of hydrogen sulfide (H_2_S) in the bioreactor resulting in the following reaction: Cys‐S‐S‐Cys + H_2_S + O –> Cys‐S‐S‐S‐Cys + H_2_O (Gu et al., 2010). The presence of H_2_S in the bioreactor has been demonstrated by two different approaches, the first is the enzymatic reduction of cysteine by mammalian cells resulting in the production of H_2_S (Kamoun, 2004; Kimura, 2011) and the second source of H_2_S was from the feed media (Kshirsagar et al., 2012). In the present work, an investigation was carried out to determine if the H_2_S in the bioreactor could be stripped off and lead to a reduction in TSB levels as was demonstrated by Becker and Christensen (2005). The gas flow rates of CO_2_ were manipulated for 3 cell culture runs performed at the 15 mL scale. The following set points were implemented for the three culture runs: temperature set to 35.5°C, pH set to 7, nutrient feed day equal to 1, nutrient feed volume equal to 1 mL/mL, and seeding density equal to 1 cells mL^−1^/cells mL^−1^. The CO_2_ gas flow rates were ramped up from normal operation to 1.75 and 2.25 times normal operation as is shown in Supporting Information Figure S1(a). The TSB formation of these three cell culture runs are shown in Supporting Information Figure S1(b) and demonstrate a reduction in TSB formation with the increased gas sparging. Kshirsagar et al. (2016) demonstrated the primary source of H_2_S was the result of cysteine in the media feed and demonstrated that an eightfold increase in cysteine in the media resulted in TSB concentrations increasing from approximately 2% to 15%. However, in the present study no correlation was found between the cumulative feed added compared to the TSB levels. Larger adjustments to the feed volume may be required to see any observed differences as the feed volume was only adjusted by ±20% in this case study. Further work investigating the levels of amino acids in particular the cysteine concentration would be needed to verify this correlation. The pH was also found to be significant in the mechanism of trisulfide bond (Becker and Christensen, 2005; Nielsen et al., 2011). Nielsen et al. (2011) proposed the formation of trisulfides in proteins is the result of a nucleophilic attack of the sulfide ion (SH^−^) on the disulfide bond of the protein resulting in the formation of a trisulfide bond. This proposed formation of TSB is dependent on a number of factors including the presence of the SH^−^ and a pH at or above neutral. Analysis of the time‐series regression weights (Fig. 7b) demonstrates a high pH during the later stages of cell culture run would promote TSB formation. Furthermore, all the cell culture runs with high levels of TSB (>6%) had a high end‐point pH (pH>7) (data not shown).

### Validation of MVDA Models Upon Scale‐Up

**Figure 9:**
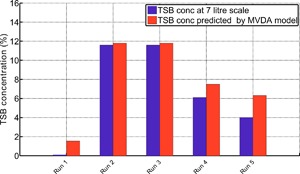
TSB concentrations predicted by the linear regression model compared to five 7 L bioreactor runs operated at various different operating conditions using the same cell line implemented by the micro‐bioreactor system. The MLR model predictions were based on the initial concentrations, feeding strategy implemented, and process set‐points defined in Table I.

Both the MLR and PLS models provided unique and complementary insights into the primary variables influencing the TSB concentrations, with the culture temperature and pH highlighted as the two main variables influencing the TSB formation. To ensure the predictions from these models were valid upon scale‐up, five 7 L bioreactor runs using the same cell line were set up. The selected process conditions are shown in Table I and the MVDA model predicted that these process predictions would result in runs that contained TSB concentrations from 0% to approximately 10%. These predictions were determined using Equation (1) taking the inputs of the model as the temperature, pH, and initial feed day shown in Table I. Figure 9 compares the experimentally recorded TSB concentrations against the concentration predicted by the model. A high coefficient of determination (*R* ^2^ = 0.90) was obtained that indicated good agreement between the experimental and predicted values of TSB. These accurate predictions at scale‐up demonstrate the importance of this relatively simple model to extract valuable information from a complex data set. The more complex PLS model outlines the importance of considering the time‐series pH deviations in addition to the gas flow rates that could be manipulated to minimise TSB formation in recombinant protein manufacturing. To determine if these trends and the resulting predictive model can be generalized in terms of process parameters influencing TSB formation on therapeutic proteins, it would be necessary to collect data over a wider range of cell lines and products.

## Conclusion

This work demonstrates the successful application of MVDA techniques to analyze a large complex dataset generated by a HT micro‐bioreactor system and outlined the key process parameters that influence TSB formation on an industrially relevant antibody–peptide fusion cell culture process. Two MVDA techniques were implemented to leverage significant insights from the DoE datasets generated with the Ambr^*TM*^‐15 system. The first MVDA technique applied was an MLR model that identified the key process conditions resulting in high TSB concentrations. The MLR model indicated that a high temperature set‐point (37°C) and a low pH set‐point (<6.8) combined with a late nutrient feed day (day 3 or 4) would result in high TSB concentrations equal to approximately 15%. To further scrutinize the available dataset, a PLS model was generated that complemented the MLR model findings and highlighted the temperature and pH as key process variables influencing the TSB concentration. The ability of the PLS model to not only consider set‐point changes but also the whole time‐series datasets of all the process variables proved highly relevant and highlighted a pH shift on day 6 to be significant in influencing the TSB concentration. The process conditions identified by both the MLR and PLS models were manipulated on five 7 L bioreactors to validate the predictions of the MVDA models at laboratory scale and demonstrated excellent agreement with the TSB concentrations recorded for each of the five bioreactors. The insights generated from this work enable the control limits of the key process parameters to be redefined to ensure the TSB concentration is minimized. Furthermore, the proposed MVDA approach outlined here is a universal methodology enabling root‐cause analysis of other post‐translation modifications to be easily and systematically identified.

The authors would like to thank Chris Sellick, Neil Birkett, Karen Dickson, and Andrew Smith from MedImmune for their valuable help with analytics on this project. Furthermore the authors would like to thank Tarik Senussi for providing the cell line. This research is associated with the joint UCL‐MedImmune Centre of Excellence for predictive multivariate decision‐support tools in the bioprocessing sector and financial support from MedImmune and UCL for S. Goldrick is gratefully acknowledged. Furthermore support from the EPSRC is also greatly appreciated (EP/I033270/1).

## Supporting information

Additional supporting information may be found in the online version of this article at the publisher's web‐site.

**Figure S1**. Analysis of three cell culture runs operated at three different CO_2_ sparging rates with (a) the cumulative CO_2_ gas flow rates (*F*_CO2_) and (b) the TSB concentration of the three cell culture runs.
