# Supplementary material for: Advanced multivariate data analysis to determine the root cause of trisulfide bond formation in a novel antibody–peptide fusion
Source: Biotechnol Bioeng. 2017 Jun 5;114(10):2222–34. doi: 10.1002/bit.26339 (PMC5600124; doi:10.1002/bit.26339)
Supplement: Supplementary file 1 — Figure S1. Analysis of three cell culture runs operated at three different CO2 sparging rates with (a) the cumulative CO2 gas flow rates (F CO2) and (b) the TSB concentration of the three cell culture runs. [file BIT-114-2222-s001.docx]

**Supplementary Material**


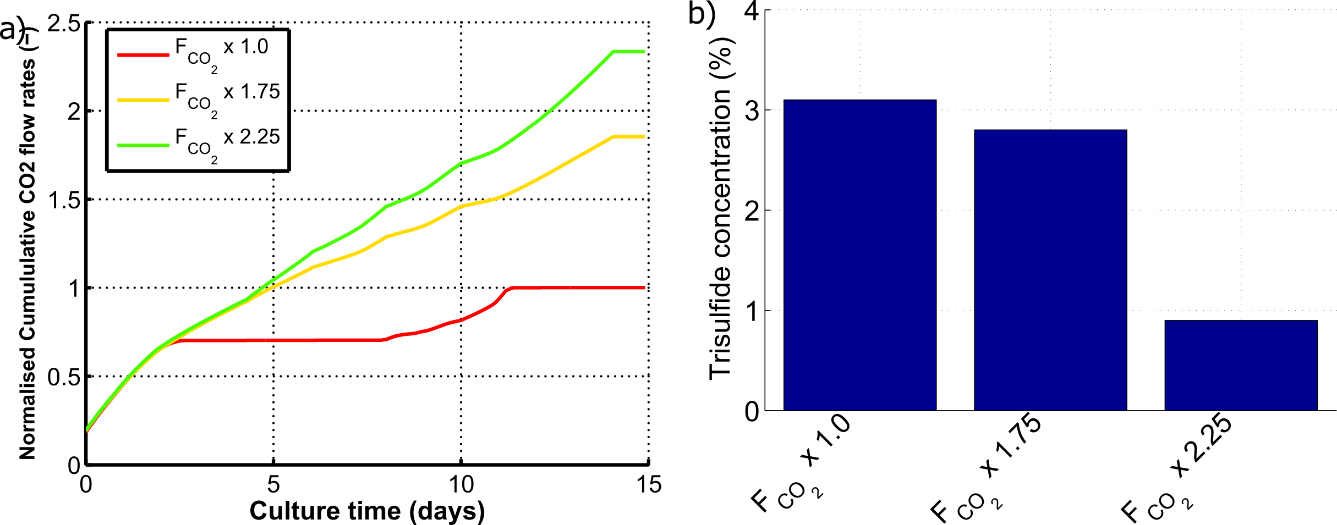


**Supplementary Figure 1:** Analysis of three cell culture runs operated at three different CO_2_ sparging rates with a) the cumulative CO_2_ gas flow rates (F_CO2_) and b) the TSB concentration of the three cell culture runs.
